# Supplementary material for: The Effect of Cropping Systems on the Dispersal of Mycotoxigenic Fungi by Insects in Pre-Harvest Maize in Kenya
Source: Insects. 2024 Dec 16;15(12):995. doi: 10.3390/insects15120995 (PMC11677841; doi:10.3390/insects15120995)
Supplement: Supplementary file 1 [file insects-15-00995-s001.zip › insects-3324634-supplementary.pdf]

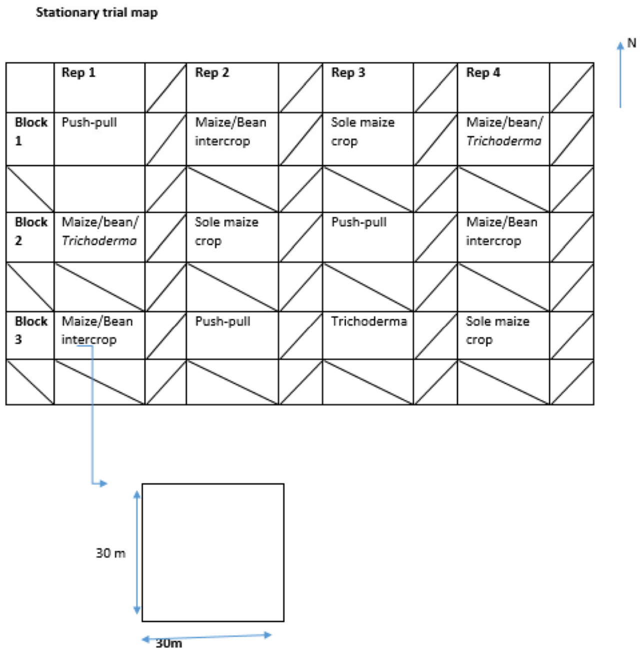

Figure S1. The generic experimental design for the on-station trials in Kibos in Kisumu and Kambi ya Mawe in Makueni County.

NOTES

- The trial has four treatments:

  Maize/bean intercrop
  Push-pull- maize/ Desmodium intercrop, with three rows of Napier on the edges
  Maize/bean intercrop and treated with *Trichoderm*a and
  Sole maize crop
- Plot sizes: 30 x 30 metres, paths 2 Metres
